# Effect of CB2 Stimulation on Gene Expression in Pediatric B-Acute Lymphoblastic Leukemia: New Possible Targets

**DOI:** 10.3390/ijms23158651

**Published:** 2022-08-03

**Authors:** Francesca Punzo, Maura Argenziano, Chiara Tortora, Alessandra Di Paola, Margherita Mutarelli, Elvira Pota, Martina Di Martino, Daniela Di Pinto, Maria Maddalena Marrapodi, Domenico Roberti, Francesca Rossi

**Affiliations:** 1 Department of Woman, Child and General and Specialist Surgery, Via Luigi De Crecchio 4, 80138 Naples, Italy; francesca.punzo@unicampania.it (F.P.); maura.argenziano@unicampania.it (M.A.); chiara.tortora@unicampania.it (C.T.); alessandra.dipaola@unicampania.it (A.D.P.); elvira.pota@policliniconapoli.it (E.P.); martina.dimartino@policliniconapoli.it (M.D.M.); daniela.dipinto@policliniconapoli.it (D.D.P.); mariamaddalena.marrapodi@studenti.unicampania.it (M.M.M.); domenico.roberti@unicampania.it (D.R.); 2 Istituto di Scienze Applicate e Sistemi Intelligenti “Eduardo Caianiello” ISASI-CNR, Via Campi Flegrei 34, 80078 Pozzuoli, Italy; margherita.mutarelli@cnr.it

**Keywords:** acute lymphoblastic leukemia, SUP-B15 cell line, endocannabinoid system, RNA sequencing, CB2 receptors

## Abstract

Acute lymphoblastic leukemia type B (B-ALL) is the most common kind of pediatric leukemia, characterized by the clonal proliferation of type B lymphoid stem cells. Important progress in ALL treatments led to improvements in long-term survival; nevertheless, many adverse long-term consequences still concern the medical community. Molecular and cellular target therapies, together with immunotherapy, are promising strategies to overcome these concerns. Cannabinoids, enzymes involved in their metabolism, and cannabinoid receptors type 1 (CB1) and type 2 (CB2) constitute the endocannabinoid system, involved in inflammation, immune response, and cancer. CB2 receptor stimulation exerts anti-proliferative and anti-invasive effects in many tumors. In this study, we evaluated the effects of CB2 stimulation on B-ALL cell lines, SUP-B15, by RNA sequencing, Western blotting, and ELISA. We observe a lower expression of CB2 in SUP-B15 cells compared to lymphocytes from healthy subjects, hypothesizing its involvement in B-ALL pathogenesis. CB2 stimulation reduces the expression of *CD9*, *SEC61G*, *TBX21,* and *TMSB4X* genes involved in tumor growth and progression, and also negatively affects downstream intracellular pathways. Our findings suggest an antitumor role of CB2 stimulation in B-ALL, and highlight a functional correlation between CB2 receptors and specific anti-tumoral pathways, even though further investigations are needed.

## 1. Introduction

Leukemia is the most common pediatric tumor, and, despite a good survival rate, it is responsible for almost 30% of cancer deaths in children [1]. Different kinds of leukemias affect children and adolescents, with the most diffused subtype being acute lymphoblastic leukemia (ALL), characterized by the clonal proliferation of lymphoid stem cells, principally with a B-cell phenotype (more than 80%) [2]. ALL surely represents a very heterogeneous disease and current treatment modalities, applied since the 1990s and beyond, stratified ALL patients into risk groups and tailored treatment accordingly [3,4]. During the last few decades, pediatric ALL cure rates improved to 90%, with a parallel progress in event-free and 5 year survival rates. This important clinical success principally derives from patients classification into risk groups related to prognostic factors and treatment intensity, adjusted based on these risk categories [5]. High-risk B-ALL patients show a very high medical need, as well as many adverse long-term consequences, which is very concerning to the medical community. ALL is still responsible for a third of cancer-related deaths in children, for inevitable late effects due to cancer itself and therapies, and a compromised lifestyle (poor nutrition and physical inactivity) [6,7]. Modern therapeutic protocols for ALL reached the highest possible effectiveness, according to what is reported in the literature and in clinical practice [8,9,10]. Even though little can be done to further improve outcomes for ALL patients without increasing toxic effects, stronger efforts are necessary to identify new strategies for the group of high-risk patients who relapse or are primary refractory. To combine or replace classical therapeutic strategies with molecular- and cellular-targeted therapy and immunotherapy could help moving beyond the classical cytotoxic chemotherapy [8,11,12]. The antineoplastic role of cannabinoids in malignancy of the immune system, [13,14] as well as in many other tumors, i.e., osteosarcoma, is well documented [15,16]. Cannabinoids derive from the Cannabis plant, and interact with the cannabinoid receptors CB1 and CB2, principally expressed in the central nervous system and in peripheral and immune cells, respectively [17]. These receptors, together with their specific ligands (endocannabinoids) and the enzymes involved in their own synthesis and degradation, constitute the endocannabinoid system (ECS). ECS is involved in many biological functions, such as pain management, regulation of appetite, control of bone metabolism [18,19], and, noteworthily, it modulates both inflammatory processes and immune response [20]. Several authors proposed ECS as anticancer target for different neoplasms [21]; in particular, a proper stimulation of CB2 receptors is responsible for counteracting tumor growth and progression [22]. CB2 receptors modulates migration, immune functions, and cytokine production in immune cells, but it is also expressed in different types of cancer cells (breast cancer, pancreatic cancer, T-cell ALL) [23,24,25]. CB2 receptors can mediate anticancer effects, avoiding the psychiatric side effects normally associated with CB1 receptors [26,27]. In our previous studies, we demonstrated the anti-proliferative, pro-apoptotic, and anti-invasive effects of CB2 receptors selective stimulation in both T-ALL and osteosarcoma in vitro [15,28]. Considering the promising pharmacological activity attributed to CB2 receptors, we tested the effects of its selective stimulation on B-ALL cell lines, SUP-B15 cells. These cells are an interesting in vitro model for our purpose because they are a Philadelphia chromosome-positive B-ALL cell line derived from an 8-year-old high-risk patient, resistant to imatinib, a tyrosine kinase inhibitor [29]. Hence, they are good representatives of the category of patients with the worst prognosis and for whom more effective therapies are needed. We demonstrate the involvement of ECS in this neoplasm and highlight the possibility to target it to arrest growth and progression of B-ALL.

## 2. Results

### 2.1. CB2 Receptor Expression in SUP-B15 Cell Line

**Figure 1:**
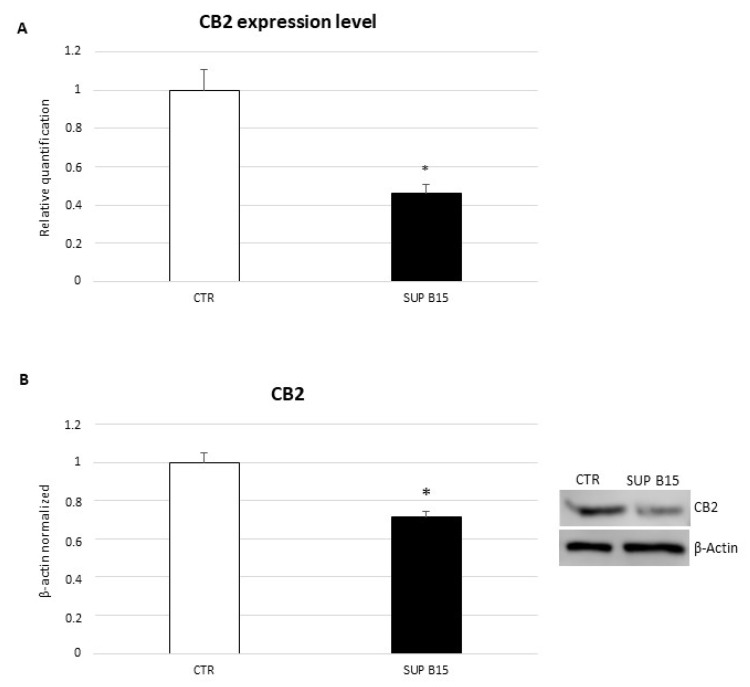
CB2 gene (**A**) and protein (**B**) expression levels in SUP-B15 cell line compared with CTR lymphocytes. For real-time PCR (**A**), CB2 mRNA expression levels of SUP-B15 cell line are compared with healthy controls-derived lymphocytes (CTR). Results are normalized for the housekeeping gene β-actin, and shown as mean ± S.D. of three independent experiments * indicates *p* ≤ 0.05 compared to CTR. Western blot (**B**) was performed starting from 20 μg of total lysates. The most representative images are displayed. The proteins were detected using Image Studio Digits software, and the intensity of immunoblots compared to the control, taken as 1 (arbitrary unit), were quantified after normalizing with respective loading controls for the housekeeping protein β-actin. Histogram shows CB2 receptor expression levels as the mean ± S.D. of three independent experiments. A t-test was used for statistical analysis. * indicates *p* ≤ 0.05 compared to CTR.

We studied the expression of CB2 receptors in the SUP-B15 cell line by evaluating mRNA and protein expression levels, with real-time PCR and Western blotting, respectively. Both real-time PCR and Western blotting show a strong and statistically significant reduction in CB2 receptor expression levels in the SUP-B15 cell line in comparison with lymphocyte primary culture obtained from healthy donors (Figure 1A,B). This result, confirming the expression of CB2 receptors in SUP-B15 cells, as well as in other peripheral immune cells, is preliminary to the subsequent treatments with selective agonist and inverse agonist, JWH-133 and AM630, respectively, to the receptor itself.

### 2.2. Effect of CB2 Stimulation on Gene Expression: RNA-Sequencing Analysis

Using RNA sequencing, we identify four key markers of cancer development and progression differentially expressed in SUP-B15 cells before and after the pharmacological stimulation of CB2 receptors. We observe a lower transcription of *CD9* (−2.4-folds), *SEC61G* (−1.6-folds), *TBX21* (−1.2-folds), and *TMSB4X* (−1.1-folds) genes, after treatment with JWH-133, while no effects are apparent after AM630 administration (Table 1). This result indicates that the altered observed mRNA expression is exclusively due to the selective stimulation of the CB2 receptors. Each of the analyzed genes plays a specific role in cancer and is downstream related to other genes, proteins, and pathways. We identify several correlations of interest and describe them below, in order to unravel and better understand the antitumor efficacy of CB2 receptors.

### 2.3. Effects of CB2 Receptor Stimulation on CD9-RAC1 Signaling Pathway

**Figure 2:**
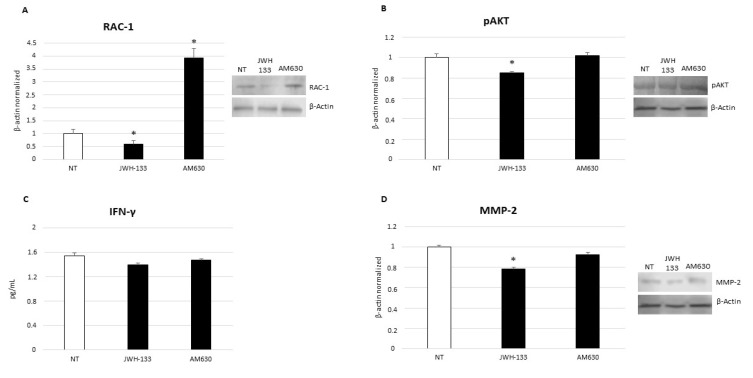
RAC-1 (**A**), pAKT (**B**), and MMP-2 (**D**) protein expression levels in SUP-B15 cell line after treatment with JWH-133 and AM630 compared with non-treated control (NT), determined by Western blot, starting from 20 μg of total lysates. The most representative images are displayed. The proteins were detected using Image Studio Digits software, and the intensity of immunoblots compared to the control, taken as 1 (arbitrary unit), were quantified after normalizing with respective loading controls for the housekeeping protein β-actin. Histogram shows RAC-1, pAKT, and MMP-2 protein expression levels as the mean ± S.D. of three independent experiments. A t-test was used for statistical analysis. * indicates *p* ≤ 0.05 compared to NT. (**C**) IFN-γ concentrations (pg/mL) in SUP-B15 cells after treatment with JWH-133 and AM630 compared with non-treated control (NT), determined by enzyme-linked immunosorbent assay (ELISA Assay). Histogram shows IFN-γ concentrations as the mean ± S.D. of three independent experiments. The cytokine concentration was determined on a standard concentration curve, according to the manufacturer’s instructions. A t-test was used for statistical analysis. * indicates *p* ≤ 0.05 compared to NT.

The CD9-RAC1 signaling pathway plays a crucial role in B-ALL, enhancing the migration and homing of pre-B leukemic cells to bone marrow. CD9 protein induces the activation of Ras-related C3 botulinum toxin substrate 1 (RAC1) [30]. After treatment with JWH-133, the RNA sequencing analysis shows a 2.4-fold reduction in the expression of CD9 encoding gene in SUP-B15 cells. Considering this result, we performed Western blotting to also evaluate the effect of treatment on RAC1 protein and, as expected, we observe a significant reduction in its expression (Figure 2A). This result highlights a correlation between CB2 stimulation and the CD9-RAC1 signaling pathway, but the exact underlying mechanism is not known and must be clarified with further molecular studies. The observed effects are certainly only due to CB2 stimulation, since we do not observe any alteration in *CD9* gene and RAC1 protein expression levels after treatment with AM630, the selective inverse agonist for CB2 receptors.

### 2.4. Effects of CB2 Receptor Stimulation on Leukemia Cell Proliferation

Sec61 translocon gamma subunit (SEC61G) protein is over-expressed in different kinds of cancer and enhances tumor cell growth [31]. Silencing of this protein blocks AKT survival signaling, thus, playing an anticancer role. Using RNA- sequencing, we observe a statistically significant reduction in its gene expression (1.6-fold) after 12 h treatment with JWH-133. Consequently, we also evaluated the effects of JWH-133 administration on AKT activation and we observe, again, a statistically significant reduction in expression of its phosphorylated form, confirming the anticancer activity of CB2 receptor stimulation (Figure 2B). Currently, no evidence on the molecular mechanisms regulating interaction between CB2 receptors and SEC61G protein is reported, thus. making deeper investigations necessary. 

### 2.5. Effects of CB2 Receptor Stimulation on Leukemia Cell Survival

JWH-133 12 h treatment induces a significative reduction in both *TBX21* gene expression and INF-γ release in SUP-B15 cells (Figure 2C). The *TBX21* gene encodes for T-bet protein, a T-box transcription factor expressed in B cell precursors, and involved in the oncogenesis of T-bet-positive B-cell neoplasms [32]. INF-γ is a well-known pro-inflammatory cytokine, responsible for the apoptosis and differentiation of B cell precursors [33]. It is known that INF-γ normally induces the expression of the T-bet transcriptional factor, and that the proper stimulation of CB2 exerts anti-inflammatory effects. Moreover, we also evaluated the effects of CB2 receptors 12 h stimulation on cell survival by means of cytofluorimetric assays on cell viability, cell death, and apoptosis. After JWH-133 administration, we observe a reduction in viable cells percentage (Table 2), as well as an increase in early apoptotic cells (Table 3). Although not statistically significant, it reveals an initial trend towards the overall reduction in leukemic cell survival. The results we observed with these experiments are attributable only to CB2, since the treatment with AM630, the inverse agonist at the cannabinoid receptor, does not cause the same effects.

### 2.6. Effects of CB2 Receptor Stimulation on Invasion Capacity of Leukemia Cells

To investigate the effects of CB2 stimulation on the invasive capabilities of the SUP-B15 cell line, we analyzed the expression of MMP-2 protein using Western blotting, and of the *TMSB4X* gene by RNA-seq, and we observe a trend towards reduction in their expression levels after 12 h exposure to the cannabinoid JWH-133 (Figure 2D). The *TMSB4X* gene encodes for the thymosin beta 4 (Tβ4) protein, which is involved in hematopoietic stem cell function and mobilization, while the MMP-2 protein regulates cancer cell migration and invasion [34]. This result is coherent with the known regulating property exerted by the *TMBS4X* gene on MMP-2 protein expression via the Wnt/β-catenin/Lef-1 signaling pathway [35], and it is very likely only due to CB2 stimulation, since we do not observe the same reduction after AM630 treatment.

## 3. Discussion

Acute lymphoblastic leukemia (ALL) represents 25% of total pediatric cancer diagnoses, and primarily presents in patients under 20. In particular, B-cells-related ALL is the most frequently occurring type (85%) [36]. The currently available therapy for ALL guarantees a survival rate exceeding 90%, and it is not possible to obtain further improvements with just conventional chemotherapy without increasing the side effects. For these reasons, in recent years new different therapeutic approaches were proposed to increase ALL survival outcome, such as molecular target therapy with the use of tyrosine kinase inhibitors [37], BCL-2 inhibitors [38], and proteasome inhibitors [39], or immunotherapy, in particular CAR T cells, antibody-drug conjugates and bispecific antibodies [40]. The endocannabinoid (EC) neurotransmitter system has a key role in several biological processes, including cancer onset and development [27,41]. The clinical use of cannabinoids is well-validated in palliative medicine, and pharmacokinetic and safety data on their use are already available, thus, making their hypothetical introduction into the oncological clinic easier and faster. We investigated whether the specific stimulation of CB2 receptors also has anticancer effects in the B-cell ALL cell line, SUP-B15. In particular, our study aim was to describe and clarify the initial adaptive response to short and selective agonist pulse (12 h) on CB2 receptors. We successfully verify the presence of CB2 receptors on this cell line, thus, being certain of the possibility to treat with JWH-133. Interestingly, SUP-B15 cells show lower levels of the cannabinoid receptor than lymphocytes from healthy subjects, leading to hypothesize its involvement in the pathogenesis of the leukemic disease. In 2018, Lai N. Chan et al. already described the central role of CB2 receptors in preventing the malignant transformation of pre-B cells and, consequently, the onset of B-ALL, demonstrating the beneficial effects of its proper stimulation with specific agonists [42]. To understand the actual involvement of CB2 in B-ALL, we firstly checked the effects of its selective stimulation on transcription activity. Over the last 10 years, RNA sequencing (RNA-seq) protocols rapidly evolved, and the integration of next generation sequencing into clinics could certainly be a successful clinical tool [43,44]. In our study, we observe a decrease in *CD9*, *SEC61G*, *TBX21****,*** and *TMSB4X* gene transcription. These genes are all responsible for cancer onset, growth, progression, and maintenance. CD9 is a protein of the tetraspanin family, involved in both physiological and pathological events such as tumor progression and metastasis [45]. Marie-Pierre Arnaud et al. described its crucial role in enhancing pre-B leukemic lymphocytes invasion to the testis, a very common site of ALL late relapses [30]. In particular, in ALL patients they observe a CD9-mediated overexpression of the RAC1 protein, an important mediator of cell migration and tumor progression. In our study, JWH-133 reduces *CD9* gene transcription and also RAC1 protein expression, thus, possibly resulting in a limitation of invasive capabilities. The interaction between CB2 receptors and the *CD9* gene is not described in the literature, and it could be interesting to better understand the mechanisms underlying this link. On the contrary, Zhe Wang already described the influence of CB2 stimulation on the RAC1 pathway [46], supporting the idea that the observed effect on SUP-B15 migration could be due to a direct effect of CB2 stimulation on RAC1 expression, and contemporary to CD9 gene transcription reduction mediated by JWH-133. Another important aspect we investigated is the proliferative potential of cancer cells. Cancer cells show a high rate of proliferation, and SEC61G is one of the main genes involved, acting as proto-oncogene and enhancing cancer cell survival [44,47]. It is observed that by knocking it out, EGFR/AKT survival signaling, and tumor growth are inhibited [31]. To the best of our knowledge, no authors describe the interaction between SEC61G and the EC system, but in this study, we observe a reduction in the transcription of SEC61G after the selective stimulation of CB2, thus, suggesting a correlation between them. In addition, we also observe a reduction trend in the expression of the phosphorylated (or active) form of AKT after using JWH-133. This result supports what is already reported in the literature for other selective agonist at CB2 receptors, such as quinoline carboxamides, which are known to inhibit the expression and phosphorylation of the pro-proliferative kinase pathway Akt [48,49]. Considering that SEC61G inhibition blocks AKT signaling, it is very probable that in this case also, CB2 could arrest tumor cell proliferation by a dual mechanism: a direct effect on pAKT, and an indirect effect mediated by SEC61G. More efforts to understand the molecular mechanism regulating these interactions are certainly necessary. The proposed beneficial effect of CB2 stimulation in limiting SUP-B15 cell survival and proliferation is also evident in cytofluorimetric experiments. We observe a trend towards a reduction in cell viability, together with an increase in apoptotic cell percentage. Although there is no statistical significance in this result, it is very relevant considering how early it appears. Indeed, SUP-B15 cells normally have a doubling time of almost 18 h (PubMed 3162827); the administered short pharmacological pulse sorts its effects in a very rapid manner, influencing the earliest intracellular processes (mRNA transcription and protein translation). B-cell-derived neoplasms are found to express the *TBX21* gene [32], which encodes for T-bet protein and seems to have an important role in the oncogenesis of this category of hematological diseases. Akira Harashima et al. demonstrate that INF-γ induces the expression of *TBX21* in monocytic leukemia cells [33], while no evidence in the literature is reported about its expression and induction in primary B cell precursors; its inducted is only observed in B cell precursors cell lines. The role of T-bet must be better clarified, but it seems to be involved in the differentiation of B cell precursors. In the past, no correlations were described between *TBX21* expression and CB2 receptor selective stimulation, nevertheless, we observe a reduction in its transcription after administering JWH-133. This result encourages the unravelling of whether the EC system could influence the activity of this transcriptional factor. After using JWH-133, we observe a dramatic reduction in INF-γ release, which normally has higher levels in ALL patients before treatment then in healthy subjects [50]. In the literature, the effects of CB2 receptor stimulation in reducing INF-γ levels are well-documented, and we too observe this very effect in our experiments. Hence, we observe a cascade event, in which CB2 selective stimulation with JWH-133 causes a reduction in INF-γ release, which, in turn, could be the cause of the reduction in *TBX21* gene induction. All together, these results suggest a beneficial effect of CB2 stimulation in limiting tumorigenesis in B-ALL. After stimulating CB2 receptors, RNA-seq also shows a reduction in *TMSB4X* gene expression, which encodes for Tβ4 protein, and is one of the most studied cell migration mediators [51]. Several authors report a tight correlation between the *TMSB4X* gene and metalloproteinases (MMPs), in particular MMP-2, principally involved in the activation of cell migration and growth. In 2016, Xiao-yu Gao described how *TMSB4X* regulates MMP-2 expression levels via the Wnt/β-catenin/Lef-1 signaling pathway influencing cell migration. MMPs have a key role in hematopoietic stem cell mobilization, as well as in cancer-allowing progression and invasion [52]. Even though their role in ALL is not yet very clear, they could represent a good target for both prognosis and therapy, since they play a key role in tumor angiogenesis and metastasis [53]. In our experiments, we obtain a reduction in both *TMBS4X* gene and MMP-2 protein expression levels. While in the literature the direct effect of cannabinoids in reducing the expression of MMP-2, also attenuating invasion in leukemic cells [54], is well-documented, there are no data regarding the interaction between the *TMBS4X* gene and CB2 receptors. So, it could be interesting to investigate the reasons behind the reduction in gene expression after treatment with JWH-133. Another noteworthy aspect of our study is that all the observed effects are due only to CB2 selective stimulation; indeed, after the administration of AM630, an inverse agonist at CB2 receptors, there is no relevant change, thus, meaning that when CB2 receptors are blocked, there are no beneficial and curative effects.

## 4. Materials and Methods

### 4.1. Cell Culture

SUP-B15 cell line was purchased from ATCC and cultured in Iscove-modified Dulbecco medium, supplemented with 20% fetal bovine serum (FBS), 100 U/mL penicillin, 100 U/mL streptomycin, and 2 mM L-glutamine. This cell line derives from malignant cells from bone marrow of an 8 year old Philadelphia chromosome-positive B-ALL patient. These cells were grown in suspension in 100 mm dishes. After a week, SUP-B15 cells were washed and counted on a microscope using a Burker Haemocytometer (Waldemar Knittel Glasbearbeitungs GmbH, Braunschweig, Germany) and 1.0 × 10^6^ cells per well were plated in a 6-well cell culture multiwell for treatment with JWH-133 [100 nM] and AM630 [10 µM]. After 12 h exposure, cells were harvested for RNA sequencing and protein extraction, and supernatants were destined for ELISA assay. Lymphocytes were obtained from PBMCs by Ficoll density gradient centrifugation from venous blood of three healthy subjects (median age 6.8 ± 2 years), and were cultured in Roswell Park Memorial Institute (RPMI) 1640 medium, supplemented with 10% FBS, 2 mM L-glutamine, and 100 U/mL penicillin/streptomycin, with mitogen-induced stimulation by phytohemagglutinin (PHA). Lymphocytes were cultured in suspension in T25 Flasks. They were harvested at 80% of confluence for protein extraction. Both SUP-B15 cells and lymphocytes were cultured at 37 °C in a humidified atmosphere with 5% CO_2_. Healthy donors were enrolled in Department of Women, Children, and General and Specialist Surgery of University of Campania “Luigi Vanvitelli”. All procedures performed in this study were in accordance with the Helsinki Declaration of Principles, the Italian National Legislation, and the Ethics Committee of the University of Campania Luigi Vanvitelli, which formally approved the study (Identification code 266, 18 September 2020). Written informed consent was obtained from parents, and assent was acquired from children before any procedures.

### 4.2. Drugs and Treatments

Treatments were performed using JWH-133 (potent CB2 selective agonist) and AM630 (CB2 inverse agonist). Both drugs were purchased from Tocris as powder, and then dissolved in PBS containing dimethyl sulfoxide (DMSO). DMSO final non-toxic concentration on cell cultures was 0.01%. SUP-B15 cells were treated with JWH-133 [100 nM] and AM630 [10 μM] for 12 h, and contemporary non-treated cultured cells were maintained in incubation media with or without vehicle (DMSO 0.01%). Both JWH-133 and AM630 were used to the concentration defined through concentration-response experiments and producing the strongest effect without altering cells viability.

### 4.3. RNA Isolation and Real Time PCR

The total RNA was extracted using GeneElute Mammalian Total RNA Miniprep Kit (Sigma-Aldrich, Milano, Italy), according to the manufactures’ instructions for both cDNA transcription and RNA sequencing. EasyScript cDNA Synthesis Kit (abm, Canada) was used to synthesize from approximately 500 ng mRNA, the first strand cDNA. The transcript level of CB2 was detected by RT-qPCR using a CFX96 real-time PCR system (Bio-Rad, California, USA) and using I-Taq Universal SYBR Green Master Mix (Bio-Rad). The cycling conditions were 10 min at 95 °C (initial denaturation), followed by 40 cycles of 15 s at 94 °C (denaturation) and 1 min at 68 °C (annealing/extension/data collection). The β-actin gene served as the reference gene for the normalization of the RT-qPCR products. Assay linearity and efficiency were tested over dilutions of input cDNA spanning five orders of magnitude. Assays were performed in technical triplicate. The dissociation curve analysis of amplification products was performed at the end of each PCR reaction, to confirm the specificity of the amplification. The 2^−ΔΔCt^ method was used to analyze data and to obtain the relative gene expression compared to control.

### 4.4. RNA-Sequencing Library Preparation and Sequencing

Total RNA was quantified using the Qubit 2.0 fluorimetric assay (Thermo Fisher Scientific, Waltham, MA, USA). Libraries were prepared from 100 ng of total RNA using the QuantSeq 3’ mRNA-Seq Library Prep Kit FWD for Illumina (Lexogen GmbH, Wien, Austria). Quality of libraries was assessed using screen tape high-sensitivity DNA D1000 (Agilent Technologies, Santa Clara, CA, USA). Libraries were sequenced on a NextSeq 500 system (Illumina Inc., San Diego, CA, USA) using a single read 75 cycles strategy.

### 4.5. RNA-Sequencing Data Analysis

Illumina NovaSeq base call (BCL) files were converted in fastq file through bcl2fastq (http://emea.support.illumina.com/content/dam/illumina-support/documents/documentation/software_documentation/bcl2fastq/bcl2fastq2-v2-20-software-guide-15051736-03.pdf, accessed on 14 November 2017; version v2.20.0.422). Sequence reads were trimmed using bbduk software (https://jgi.doe.gov/data-and-tools/bbtools/bb-tools-user-guide/usage-guide/-bbmap, accessed on 14 November 2017; suite 37.31) to remove adapter sequences, poly-A tails, and low-quality end bases (regions with average quality below 6). Alignment was performed with STAR (version 2.6.0a) [55] on hg38 reference assembly. The expression levels of genes were determined with htseq-count 0.9.1 using the Ensembl gene model (release 90, 22 August 2017). Differential expression analyses were performed using edgeR on genes with more than 1 CPM in more than the minimum number of samples belonging to one condition minus 1 and less than 20% of multi-mapping reads, simultaneously, and genes with FDR below 0.05 were selected as differential expressed. Principal component analysis was performed on log2(CPM), after filtering out genes with average raw counts across the dataset of less than 5 [55,56] (For details, see Supplementary Table S1).

### 4.6. Protein Extraction and Western Blotting

Proteins were isolated from SUP-B15 cells using RIPA lysis and extraction buffer (Millipore, Burlington, MA, USA), according to the manufacturer’s instructions. To quantify the protein concentration, the Bradford dye-binding method was used (Bio-Rad, Hercules, CA, USA). CB2, RAC-1, pAKT, and MMP-2 proteins were revealed in cell total lysates by Western blotting. Twenty micrograms of denatured protein were loaded. Membranes were incubated overnight at 4 °C with rabbit polyclonal anti-CB2 (1:500 dilution; Elabscience, Houston, TX, USA), mouse polyclonal anti-RAC1 (1:1000 dilution; Invitrogen, Waltham, MA, USA), mouse polyclonal anti-pAKT (1:1000 dilution; Elabscience), mouse polyclonal anti-MMP2 (1:1000 dilution; abcam, Cambridge, UK), and then with the relative secondary antibody for 1h RT. Reactive bands were detected by chemiluminescence (Immobilion Western Millipore, Burlington, Massachusetts, USA) on a C-DiGit blot scanner (LI-COR Biosciences, Lincoln, NE, USA). A mouse monoclonal anti-β-actin (1:500 dilution; Santa Cruz Biotechnology, Dallas, TX, USA) was used as housekeeping protein to verify the protein loading. Images were captured, stored, and analyzed using “Image studio Digits” software (version 5.0, LI-COR, Lincoln, NE, USA).

### 4.7. Enzyme-Linked Immunosorbent Assay (ELISA)

ELISA was performed to determine IFN-γ concentration in cell cultures supernatants, using commercially available Human ELISA Kits (Invitrogen by Thermo Fisher, Waltham, MA, USA), according to the manufacturer’s instructions. Briefly, the microplates were coated with monoclonal antibodies specific to the cytokine. Standards and supernatants were pipetted into the wells of the microplate, and were run in duplicate. After the plate was washed, enzyme-linked polyclonal antibodies specific for IFN-γ were added to the wells. The reaction was revealed by the addition of the substrate solution. The optical density was measured at a wavelength of 450 nm using the Tecan Infinite M200 (Tecan Group Ltd., Männedorf, Switzerland) spectrophotometer. Cytokine concentrations (pg/mL) were determined against a standard concentration curve.

### 4.8. Count and Viability Assay

SUP-B15 cells count and viability were evaluated after 12 h of JWH-133 and AM630 exposure with the Muse cell analyzer machine with Count & Viability Assay Kit. The Muse Count & Viability reagent differentially stains viable and non-viable cells based on their permeability to the two DNA binding dyes present in the reagent. Fifty microliters of cell suspension (1 × 10^5^ cells/mL) was mixed with 450 μL of the Muse Count & Viability reagent and incubated for 5 min. at room temperature. The results, automatically displayed, were analyzed with Muse 1.4 analysis software for data acquisition and analysis.

### 4.9. Annexin V & Dead Cell Assay

SUP-B15 cell apoptosis was evaluated by a fluorometric assay with the Muse cell analyzer machine with the Annexin V & Dead Cell Assay Kit. Test was performed after 12 h exposure to JWH-133 and AM630. The Muse Annexin V & Dead Cell Assay utilizes annexin V to detect phosphatidylserine (PS) on the external membrane of apoptotic cells. A dead cell marker, 7-amino-actinomycin D (7-AAD), is also used as an indicator of cell membrane structural integrity. Briefly, 100 μL of a cell suspension (1 × 10^5^ cells/mL) was mixed with 100 μL of the Muse Annexin V & Dead Cell Reagent and incubated for 20 min. at room temperature in the dark. The results, automatically displayed, were analyzed with Muse software (version 1.4, Luminex Corporation, Austin, TX, USA) analysis software for data acquisition and analysis.

### 4.10. Statistical Analysis

Results are expressed as mean ± standard deviation (SD) of three independent replicates of the same experiment. Statistical analysis on data was performed using the Student’s *t*-test (XLSTAT by Addinsoft 2020. Boston, MA, USA) to evaluate differences between quantitative variables. A *p* value ≤ 0.05 was considered statistically significant.

## 5. Conclusions

Our findings describe the involvement of CB2 receptors in the pathogenesis of B-ALL, and also propose its stimulation as an innovative and effective anticancer strategy. In particular, this approach is a “molecular target therapy approach”, since the selective triggering of cannabinoid modulates both gene and protein expression. We identified a specific anti-tumoral signature playing a key role in the development and maintenance of tumors, speculating a protective effect of CB2 selective stimulation. Certainly, further investigations are needed to better understand the molecular and biochemical mechanisms underlying the observed interactions, but our study seems to already highlight a good and beneficial therapeutic perspective to ameliorate the outcome for high-risk B-ALL patients.

## Figures and Tables

**Table 1:** Differentially expressed genes between SUP-B15 cells after 12 h treatment with JWH-133 and non-treated cells (NT).

Differentially Expressed Genes	LogFC JWH-133 vs. NT	FDR JWH-133 vs. NT
*CD9*	−2.436	0
*SEC61G*	−1.636	0.001
*TBX21*	−1.193	0.025
*TMSB4X*	−1.134	0.031

**Table 2:** Count and viability assay on SUP-B15 cells after 12 h treatment with JWH-133.

	Total Number of Cells × 10^6^	Total Number of Viable Cells × 10^6^	% Cell Viability
**SUP-B15 NT**	1.28	1.12	88.10
**SUP-B15 JWH-133**	1.27	1.07	84.30
**SUP-B15 AM630**	1.27	1.10	86.60

**Table 3:** Annexin V and dead cell assay on SUP-B15 cells after 12 h treatment with JWH-133.

	% Live	% Early Apoptosis	% Late Apoptosis	% Dead	% TOT
**SUP-B15 NT**	86.80	5.70	0	7.50	5.70
**SUP-B15 JWH-133**	85.85	7.60	0	6.55	7.60
**SUP-B15 AM630**	87.50	4.90	0	7.58	4.90

## Data Availability

Data for this study have been deposited in NCBI’s Sequence Read Archive (SRA) with this accession number: PRJNA856625.

## Supplementary Materials

The following supporting information can be downloaded at: https://www.mdpi.com/article/10.3390/ijms23158651/s1.
