# Supplementary material for: Effect of CB2 Stimulation on Gene Expression in Pediatric B-Acute Lymphoblastic Leukemia: New Possible Targets
Source: Int J Mol Sci. 2022 Aug 3;23(15):8651. doi: 10.3390/ijms23158651 (PMC9369382; doi:10.3390/ijms23158651)
Supplement: Supplementary file 1 [file ijms-23-08651-s001.zip › ijms-1832177-supplementary.pdf]

Supplemtary Table S1

Sample Name	Total	Trimmed	% Trim.	Trim. passed	Unique Mapped	% uniq mapp	Multi mapped	Total mapped	% tot mapped	Couted	% counted on mapped	Detected genes
**NT**	2062923	5345	(0.3%)	2057578	1427503	69.4%	301762	1729265	84%	1010785	58.5%	9301
**JWH-133**	2830976	17563	(0.6%)	2813413	1669648	59.3%	364245	2033893	72.3%	1201407	59.1%	9913
**AM630**	2178700	4488	(0.2%)	2174212	1517858	69.8%	280640	1798498	82.7%	1054603	58.6%	9360
